# Depleted Myocardial Coenzyme Q10 in Cavalier King Charles Spaniels with Congestive Heart Failure Due to Myxomatous Mitral Valve Disease

**DOI:** 10.3390/antiox10020161

**Published:** 2021-01-22

**Authors:** Liselotte B. Christiansen, Maria J. Reimann, Anne Marie V. Schou-Pedersen, Steen Larsen, Jens Lykkesfeldt, Lisbeth H. Olsen

**Affiliations:** 1 Department of Veterinary and Animal Sciences, Faculty of Health and Medical Sciences, University of Copenhagen, Ridebanevej 9, 1870 Frederiksberg C, Denmark; lbc@sund.ku.dk (L.B.C.); mreimann@sund.ku.dk (M.J.R.); am.schoupedersen@sund.ku.dk (A.M.V.S.-P.); jopl@sund.ku.dk (J.L.); 2 Department of Biomedical Sciences, Faculty of Health and Medical Sciences, University of Copenhagen, Blegdamsvej 3B, 2200 Copenhagen, Denmark; stelar@sund.ku.dk; 3 Clinical Research Centre, Medical University of Bialystok, 15-089 Bialystok, Poland

**Keywords:** heart, degenerative mitral valve disease, dog, antioxidant, mitochondria

## Abstract

Congestive heart failure (CHF) has been associated with depleted myocardial coenzyme Q10 (Q10) concentrations in human patients. The aim of this study was to investigate associations between myocardial Q10 concentrations and myxomatous mitral valve disease (MMVD) severity in dogs. Furthermore, citrate synthase (CS) activity was analysed to determine if a reduction in myocardial Q10 was associated with mitochondrial depletion in the myocardium. Thirty Cavalier King Charles spaniels (CKCS) in MMVD stages B1 (*n* = 11), B2 (*n* = 5) and C (*n* = 14) according to the American College of Veterinary Internal Medicine (ACVIM) guidelines and 10 control (CON) dogs of other breeds were included. Myocardial Q10 concentration was analysed in left ventricular tissue samples using HPLC-ECD. CKCS with congestive heart failure (CHF; group C) had significantly reduced Q10 concentrations (median, 1.54 µg/mg; IQR, 1.36–1.94), compared to B1 (2.76 µg/mg; 2.10–4.81, *p* < 0.0018), B2 (3.85 µg/mg; 3.13–4.46, *p* < 0.0054) and CON dogs (2.8 µg/mg; 1.64–4.88, *p* < 0.0089). CS activity was comparable between disease groups. In conclusion, dogs with CHF due to MMVD had reduced myocardial Q10 concentrations. Studies evaluating antioxidant defense mechanisms as a therapeutic target for treatment of CHF in dogs are warranted.

## 1. Introduction

Coenzyme Q10 (Q10) is a lipid soluble substance with a chemical structure similar to vitamin K, consisting of a benzoquinone head with a poly-isoprenoid side chain [1]. It is ubiquitously distributed in cells of organisms where the majority is synthesized endogenously and a smaller fraction is supplied from the diet, including dietary supplements [2].

Q10 plays a crucial role as a mobile electron carrier in oxidative phosphorylation, which is the biochemical pathway providing adenosine triphosphate (ATP) for energy consuming processes such as contraction, relaxation and pumping of ions in the muscle. Q10 exists in equilibrium between reduced (ubiquinol) and oxidized (ubiquinone) forms with the redox couple providing potent antioxidant properties [1,3]. In muscle cells, the primary structural location of Q10 is the inner mitochondrial membrane [3]. Cardiac muscle has a high density of mitochondria compared to other types of muscle to be able to respond to the constant high-energy demand [4,5,6,7].

In human medicine, increasing severity of congestive heart failure (CHF) has been associated with myocardial Q10 depletion [8,9,10]. After two to eight months of oral supplementation with Q10, depleted concentrations of the coenzyme were restored in patients with cardiomyopathy [9]. Clinical trials have been done to investigate effects of dietary Q10 supplement on primary cardiac endpoints in people with CHF but results are conflicting [11,12]. A recent multi-center clinical trial using Q10 in patients with CHF for a period of two years showed promising results: A 43% reduction in the relative risk of cardiovascular death was reported in patients treated with Q10 compared to placebo [13]. The mechanisms by which oral Q10 supplement improved the cardiovascular outcome in this study remain unknown.

Myxomatous mitral valve disease (MMVD) is the most prevalent acquired heart disease among dogs [14,15] and a common underlying cause of CHF. The Cavalier King Charles Spaniel (CKCS) is especially prone to development of MMVD [16,17]. Interestingly, a study in human patients with MMVD at various clinical stages, showed a negative correlation between the myocardial concentration of Q10 and myocardial function in the patients [10]. 

There are, to the best of our knowledge, no previous studies regarding myocardial Q10 concentrations in dogs with MMVD. The primary aim of this study was therefore to investigate the possible correlation between the myocardial concentration of Q10 and MMVD severity. We hypothesized that CHF in dogs is associated with reduced myocardial Q10. Moreover, we hypothesized that the depleted myocardial Q10 was caused by a reduction in mitochondrial density. Citrate synthase activity was used as a surrogate marker of mitochondrial density based on the previous demonstration of a significant correlation to mitochondrial volume in skeletal muscle of healthy adult men [6] of this enzyme. 

Secondary objectives of the study were the assessment of a relationship between age and sex of the dog, respectively, as well as storage time, with myocardial Q10 concentrations and mitochondrial density. 

The finding of depleted myocardial Q10 concentrations in dogs suffering from severe MMVD may reveal a new insight to the pathogenetic mechanisms underlying the disease and potentially lead to the identification of a novel treatment target. 

## 2. Materials and Methods 

### 2.1. Dogs

The study was done in privately owned dogs at the Department of Veterinary and Animal Sciences, University of Copenhagen. The design was a balanced case-control study. Tissue samples were collected at the department between the years 2011 and 2019 when dog owners elected for euthanasia and donation of their dog for research purposes. Samples were collected following informed consent from the owner and with ethical approval from the Danish Animal Experimentation Inspectorate (Approval numbers: 2006/561-1145, 2012-15-2934-00700 and 2016-15-0201-01074).

The following inclusion criteria were used: dogs of any breed and age presenting for elective euthanasia with a cardiac examination including echocardiography performed on the same day and where tissue from the left ventricle was obtained. Dogs were excluded if they had either a history or any clinical or echocardiographic evidence of cardiac diseases other than MMVD. Dogs were excluded if their tissue samples were not clearly identifiable or if an insufficient amount of tissue was available for the analysis of myocardial Q10 concentrations. 

Before euthanasia, all dogs underwent a standardized examination protocol. Baseline characteristics on some of the dogs presenting between 2011–2016 have been published elsewhere [18,19,20,21,22,23,24] for other purposes.

### 2.2. Clinical Examination and Sample Collection

A thorough history was obtained by interviewing owners about their dog’s age, presence of clinical signs, appetite, other diseases and previous and current use of medication. Body weight (BW) and body condition score (BCS) of the dog were recorded, followed by jugular vein blood sampling, a physical examination and a transthoracic echocardiographic exam with simultaneous electrocardiogram recording. 

The jugular venous blood sample was obtained using a 21 G butterfly catheter connected to a vacutainer system. Two K_2_EDTA stabilized tubes and one serum separator clot activator tube were collected as standard. 

Standardized transthoracic echocardiographic views [25] were recorded from the right parasternal and left apical windows by one trained examiner (LHO) using Vivid echocardiographic systems (Vivid-i, Vivid E9 or Vivid E95) with a 5Sc or a 6S transducer (GE Medical systems, Brøndby, Denmark). A standardized echocardiographic protocol using two-dimensional, M-mode, Color Doppler and spectral Doppler was followed as previously described [26].

Euthanasia was performed with dexmedetomidine 0.02 mg/kg (Dexdomitor, Orion Pharma, Turku, Finland) and butorphanol 0.1 mg/kg given IV through a venous catheter. This was followed by an intravenous infusion of pentobarbitone at a dose of 150 mg/kg. After death, the whole heart was quickly and within 45 min from euthanasia excised and weighed. A transmural sample from the left ventricle, above the anterior papillary muscle was cut and placed in liquid nitrogen and transferred to −80 °C until analysis of Q10 concentration and CS activity. 

### 2.3. Clinical Classification

Dogs diagnosed with MMVD were classified as ACVIM stage B1, B2 or C according to previously described criteria [27,28]. In brief, dogs without a murmur and no evidence of cardiac disease were allocated to the control (CON) group. Heart murmur intensity was graded 1–6/6 [29]. Dogs having a characteristic left, apical systolic murmur with an intensity <3/6, echocardiographic evidence of mitral regurgitation without the presence of cardiac remodeling were classified as MMVD stage B1. 

When a characteristic murmur with an intensity of ≥3/6 was present with echocardiographic evidence of mitral regurgitation and cardiac remodeling (defined as the presence of LA/Ao ≥ 1.6 and LVEDDN ≥ 1.7), the dog was classified as MMVD stage B2. Stage C was defined as presence of current or past clinical signs compatible with chronic CHF (tachypnea, dyspnea, nocturnal restlessness and/or cough) in combination with echocardiographic findings as described for stage B2 [28]. Dogs with past clinical signs were not refractory to standard heart failure therapy in dogs.

Echocardiographic measurements were done off line by one observer (MJR) using EchoPac software (EchoPAC PC. Version 202, GE Medical Systems, Brøndby, Denmark) and measured as the average from five consecutive cardiac cycles. Left atrial and aortic diameters were measured at the right parasternal short axis view at the level of the aortic root at the first frame after closure of the aortic valve and a ratio calculated as previously described [30]. The internal diameter of the left ventricle in diastole was measured at chordae tendineae level using two-dimensional guided M-mode at the right parasternal short axis view and normalized to BW [31]. Fractional shortening was calculated from left ventricular dimensions obtained using M-mode [32] and the ejection fraction was calculated from measurements obtained using 2D right parasternal long-axis four-chamber view as previously described [33]. Mitral regurgitation severity grading was done using colour Doppler with the gain set just below the colour sparkling artefact in air and a standardized Nyquist limit between 0.62–0.82 m/s from the left parasternal apical four-chamber view and was classified as: no or mild (<20%), moderate (20–50%) and severe (>50%) based on the regurgitant jet area relative to left atrial area [26,34]. 

### 2.4. Q10 Analyses 

Myocardial tissue samples (5–8 mg wet weight) were analyzed for concentrations of reduced and oxidized Q10 using high-performance liquid chromatography (HPLC) with electrochemical detection (ECD) as previously described [35]. Intra- and inter-day precisions were below 5.5% for heart tissue, respectively. Recovery varied between 89 and 107%. 

Total Q10 was calculated as the sum of reduced and oxidized concentrations of Q10 and the oxidation rate of Q10 was calculated as the proportion (%) of oxidized Q10 relative to the total concentration of Q10. 

### 2.5. Citrate Synthase

Enzymatic activity of citrate synthase (CS), an enzyme in the tricarboxylic acid (TCA) cycle was measured using spectrophotometry. Tissue samples of 5–8 mg (wet weight) from the left ventricular wall were homogenized in 1.5 mL of 0.3 mol/L K_2_HPO_4_ with 0.05% BSA (pH 7.7) for 2 min on a Tissuelyser (Qiagen, Venlo, Limburg, The Netherlands). Fifteen microliters of 10% Triton X-100 was added, and samples were left on ice for 15 min before being stored at −80 °C for later analysis. The homogenate was diluted 50 times in a solution containing (in mmol/L): 0.4 acetyl-CoA, 0.6 oxaloacetate, 0.157 5,5′-dithiobis-(2-nitrobenzoic acid), and 39 Tris·HCl (pH 8.0). The change of 5,5′-dithiobis-(2-nitrobenzoic acid) to 5-thiobis-(2-nitrobenzoic acid) at 37 °C was measured spectrophotometrically at 415 nm [36] on an automatic analyzer (Cobas 6000, C 501, Roche Diagnostics, Mannheim, Germany). Citrate synthase activity was normalized to tissue weight. The ratio between Q10 and CS (Q10/CS) was calculated and expressed as the concentration of Q10 relative to mitochondrial density.

### 2.6. Statistical Analyses

Statistical analysis was performed using SAS software (version 9.4, SAS Institute Inc., Cary, NC, USA) and graphical presentation of data was done using GraphPad Prism 8.0 (GraphPad Prism software, La Jolla, CA, USA). A *p*-value < 0.05 was considered significant. Group data is presented as medians and interquartile range (IQR). 

Overall differences between the four disease groups (CON, B1, B2 and C) in Q10 concentrations (total Q10, ubiquinol and ubiquinone), oxidation rate (%), CS activity, Q10/CS, background characteristics (BW, Age) and with echocardiographic parameters (LA/Ao, LVEDDN, Fractional Shortening (%)) were tested using a one-way ANOVA followed by post hoc *t*-test when the overall *p*-value showed significance. The categorical parameters mitral regurgitation severity (no or mild, moderate and severe), sex (male/female) and breed (CKCS/non-CKCS) were compared between groups using Fisher’s exact test.

Simple linear regression analysis was used to evaluate if there was an association between time (years) from tissue sampling to analyses of Q10 concentration (storage time) and each of the outcome variables: Total Q10 concentration, oxidation rate, CS activity and Q10/CS Multiple regression analyses were used to evaluate associations between total Q10 concentration, oxidation rate, CS activity and Q10/CS (response variables) and the explanatory variables ACVIM group, age and sex of the dogs. Residuals were tested for homogeneity using visual inspection of residual plot, histogram and QQ plots in combination with the Shapiro–Wilks test. When appropriate, logarithmic transformation of the response variables was used to obtain homogeneity.

A backwards stepwise reduction was applied based on *p*-values. Variables were assessed only as main effects. A correlation between total Q10 and CS activity was tested using Spearman’s correlation.

Power calculations were based on human data, which compared myocardial Q10 concentrations in MMVD patients with and without heart failure with a standard deviation of 0.06 μg/mg [9]. A minimum sample size of four dogs in each group was required to detect a decrease of 30% in myocardial Q10 concentration in dogs with heart failure compared to dogs without heart failure with a power of 80% and a significance level of 0.05.

## 3. Results

Forty-five dogs were eligible for inclusion in the study. Three dogs were excluded due to the lack of sufficient material for Q10 analysis. Two dogs were excluded because of the presence of cardiac diseases other than MMVD, recognized on echocardiographic examination or necropsy. Forty dogs were allocated to the following disease groups: CON (*n* = 10), ACVIM B1 (*n* = 11), ACVIM B2 (*n* = 5) and ACVIM C (*n* = 14). All four groups were well-matched with regards to age and sex of the dogs. In total, 15 dogs were neutered (8 females, 7 males). The dogs allocated to the disease groups (B1, B2 and C) were all CKCS. The CON group consisted of breeds other than CKCS; Labrador Retriever (*n* = 1), Bernese Mountain dog (*n* = 1), German Shepard (*n* = 1), Golden Retriever (*n* = 1), Poodle (*n* = 1), Danish-Swedish farm dog (*n* = 1) and mixed breeds (*n* = 4). Concordantly, the CON group had significantly higher BW compared to each MMVD group. Table 1 summarizes the basic characteristics of the dogs in each disease group.

In group B1, two dogs were treated with an angiotensin converting enzyme inhibitor (ACE-i) and one dog was treated with gabapentin. One dog in stage B2 was treated with meloxicam, gabapentin, tradolan and furosemide due to syringomyelia. In group C dogs, 12 out of 14 dogs received cardiac treatments in various combinations of the following drugs: furosemide (*n* = 5), torasemide (*n* = 1), phosphodiesterase-3-inhibitor (*n* = 5), ACE-i (*n* = 4), digoxin (*n* = 1), spironolactone (*n* = 1). One dog in group C received clonazepam for treatment of non-cardiac disease. In the CON group, two dogs received medical treatment consisting of methyl prednisolone (*n* = 1) and gabapentin (*n* = 1).

**Figure 1:**
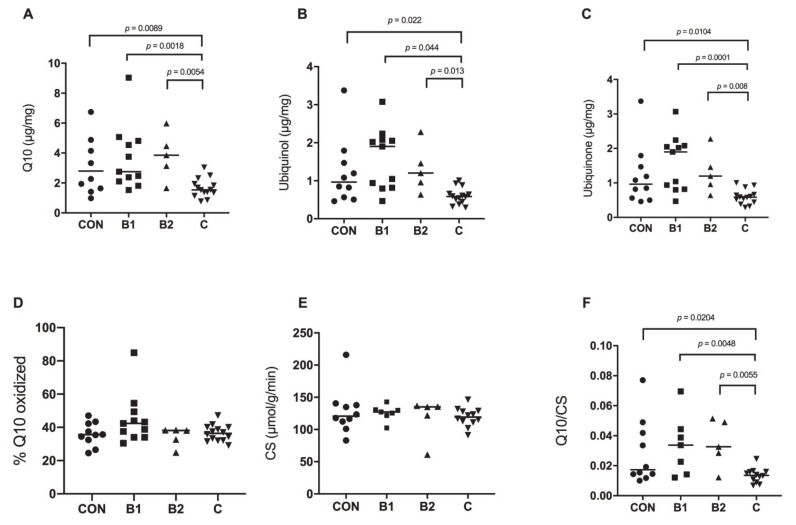
Myocardial concentrations of coenzyme Q10 and citrate synthase (CS) activity in control dogs with no evidence of heart disease (CON, *n* = 10), and in CKCS with MMVD in stage B1 (*n* = 11), stage B2 (*n* = 5) and stage C with CHF (*n* = 14). Horizontal lines indicate median values. (**A**) Total myocardial Q10, (**B**) Ubiquinol, the reduced form of Q10, (**C**) Ubquinone, the oxidized form of Q10. (**D**) Oxidation ratio (% of total Q10 that is oxidized). (**E**) Citrate Synthase activity, (**F**) Ratio between Q10 and citrate synthase activity in the disease groups.

The median time from euthanasia to analysis of the myocardial Q10 concentration was 3.5 years (IQR: 2.4–6.6). An overall significant difference was found among the four disease groups in the myocardial concentrations of total Q10 (*p* = 0.0035), ubiquinol (*p* = 0.029) and ubiquinone (*p* = 0.0008), respectively (Figure 1A–C). Post-hoc analysis revealed a significantly lower concentration of total Q10 in dogs with CHF (stage C) compared to each of the other groups: CON dogs (*p* = 0.0089), B1 dogs (*p* = 0.0018) and B2 dogs (*p* = 0.0054). The oxidation rate of Q10 was not statistically different among the groups (overall *p* = 0.076, Figure 1D). Ubiquinol was lower in stage C dogs compared to each of the other groups: CON (*p* = 0.022), B1 (*p* = 0.044) and B2 (*p* = 0.013, Figure 1B) and the same held true for ubiquinone with a significantly lower concentration found in dogs with stage C disease compared to CON (*p* = 0.0104), B1 (*p* = 0.0001) and B2 (*p* = 0.008) dogs (Figure 1C). 

Myocardial CS activity, estimating the mitochondrial density, was similar in dogs from each group (overall *p* = 0.79, Figure 1E). The ratio between Q10 and CS showed an overall statistically significant difference in Q10/CS among the groups (*p* = 0.0077). Post-hoc analysis showed a statistically significant lowering of Q10/CS in dogs with stage C disease compared to CON (*p* = 0.0204), B1 (*p* = 0.0048) and B2 (*p* = 0.0055), respectively (Figure 1F).

Male and female dogs did not differ with regards to myocardial Q10 concentrations (total Q10, ubiquinol and ubiquinone), oxidation rate, CS activity or Q10/CS.

**Figure 2:**
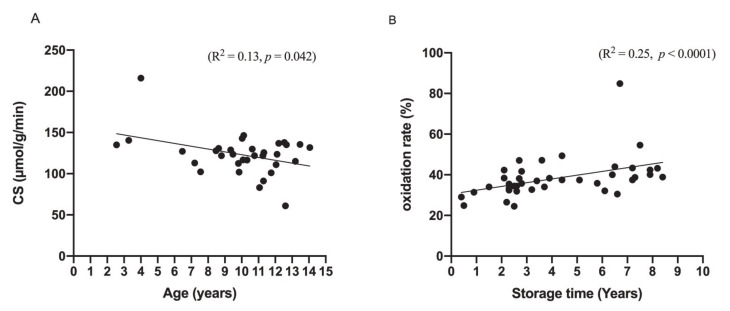
Significant associations between (**A**) myocardial citrate synthase (CS) activity and age of the dogs (*n* = 34) and (**B**) between oxidation rate and storage time of the tissue from tissue sampling to analysis of Q10 concentrations. *p*- and R^2^ values are from simple linear regression analyses.

Univariate regression analysis showed a significant association between CS activity and the dogs’ age (*p* = 0.042, R^2^ = 0.13, Figure 2A) but age was not associated with total Q10, ubiquinol, ubiquinone, oxidation rate or Q10/CS. 

The storage time of tissue (years from tissue sampling to analysis of the myocardial Q10 concentration) was shown not to be associated with the total Q10 concentration, CS activity or Q10/CS. A correlation between storage time and the oxidation rate of myocardial Q10 (*p* < 0.0001, R^2^ = 0.25) was seen (Figure 2B). No significant correlation between the myocardial concentration of total Q10 and myocardial CS activity was observed. 

The multivariate regression analysis confirmed the effect of ACVIM group on the myocardial concentration of Q10 (total, ubiquinol and ubiquinone) and on the Q10/CS ratio in the dogs. An association between age of the dog and the myocardial CS activity was also shown in the multivariate analysis. 

## 4. Discussion

We investigated myocardial Q10 concentrations in tissue samples from CKCS diagnosed with MMVD in stages B1, B2 and C according to the ACVIM guidelines and compared with a CON group of dogs with no evidence of heart disease. 

The main finding of the study was reduced concentrations of myocardial Q10 in CKCS diagnosed with MMVD in stage C compared to each of the other groups. The mitochondrial density in the myocardial tissue samples, expressed by the enzymatic activity of CS, was preserved in all stages of MMVD but declined with increasing age of the dogs. 

The results in this study in dogs is in agreement with a number of studies done in human patients with cardiomyopathy or ischemic heart disease, showing depletion of Q10 in myocardial tissue from patients diagnosed with CHF with a variety of underlying etiologies [8,9,10]. Of particular interest in relation to our results is one study showing a significant association between decreasing myocardial concentrations of Q10 with increasing severity of CHF in human patients with MMVD [10]. Our results support the hypothesis that was generated by Karlsson et al. [10], that Q10 depletion may be a relevant pathogenic factor in the progression of CHF when MMVD is the underlying cause. 

Q10 is an over-the-counter nutraceutical that comes in several different formulations and with a proven high safety in humans [13,37,38] and dogs [39]. Currently, Q10 is used as an oral supplement in dogs with CHF but its effects are solely anecdotal. The rationale behind investigating Q10 depletion in the myocardium of canine patients with heart disease is the findings in human patients where depleted myocardial Q10 was restored with the use of oral supplementation of Q10 [9]. Studies have shown clinical improvements when Q10 was used as add-on therapy to standard cardiac medication in human patients with CHF [40,41,42]. 

The inner mitochondrial membrane is the structural anchor for Q10 in cardiac muscle cells and after finding depleted myocardial Q10 concentrations in the dogs with CHF, we hypothesized that the low myocardial Q10 concentrations were associated with loss of mitochondrial density. Our finding of preserved CS activity in all groups does not suggest that changes in mitochondrial density underlie the myocardial Q10 depletion. Previous studies have investigated CS activity in the myocardium of human patients and animal models of CHF. Some studies demonstrated unchanged CS activity in the presence of CHF [8,43,44], whereas others have shown significantly reduced CS activity in patients with CHF compared to control subjects, indicating a reduction in mitochondrial density [45,46,47]. Notably, our finding of preserved CS activity in dogs with MMVD was in good agreement with two studies done in human patients with MMVD, demonstrating preserved CS activity [10,48]. This indicates that in the presence of primary valvular heart disease, a reduction of mitochondrial density does not seem to play an important role in disease progression. The deviating results from studies in patients with CHF of causes other than valvular disease may be explained by the different etiopathogenetic mechanisms underlying CHF and differences in the presence of comorbidities and use of concomitant medications. 

While CS activity was not associated with presence and severity of MMVD in the dogs, we found a negative correlation between CS activity and age of the dogs. Furthermore, the literature represents deviating results from studies that are mainly done in mice and rat models of the senescent heart, as reviewed by Boengler et al. [49]. It is the general perception that mitochondrial dysfunction, comprising changes in both functional and structural characteristics, occurs during the ageing process in myocardial cells [50]. We analysed CS activity as the only measure of mitochondrial quantity. The presence of, for example, replacement fibrosis was not within the scope of this work but is of high relevance to interpret the finding of impaired CS activity. Replacement fibrosis occurs in the myocardium of aging dogs [51] and this would reduce measures of CS activity correlated to tissue weight in myocardial tissue samples [52]. Studies need to be done in the future to understand in detail the role of age-related changes in mitochondrial mass in canine myocardium as this may be a relevant therapeutic target. In rats, age-related reductions of myocardial mitochondrial mass were successfully reversed and cardiac hypertrophy attenuated with the use of ACE-inhibitors during aging [53].

The reduced form of Q10, ubiquinol, has potent antioxidant properties and in conditions with oxidative stress, such as aging and chronic diseases, the proportion of total Q10 that is oxidized increases [54,55]. We found oxidation ratios of Q10 in the myocardial tissue samples that were comparable to previous reports from healthy dogs [35] and humans [56]. It is generally accepted that oxidative stress from increased ROS production occurs in CHF [57] and increased levels of circulating biomarkers reflecting oxidative stress have previously been suggested to be associated with severe MMVD in CKCS [58]. Nevertheless, there was no significant association between MMVD severity and oxidation ratio of Q10 in the present study. On the other hand, we found a significant correlation between storage time and oxidation ratio of Q10 in myocardial tissue samples, suggesting that oxidation may have occurred in the samples ex vivo. Importantly, the total myocardial Q10 concentrations (i.e., sum of oxidized and reduced fractions) were not associated with storage time in the dog samples but only with disease severity.

Some limitations apply to this study, one being the risk of drug interactions with myocardial Q10 concentrations. The use of cardiac medication in dogs with chronic heart failure is an unavoidable, but limiting, factor. The best described drug-interaction with Q10 in muscle is with statins, which is a frequently prescribed class of drugs for the lowering of low density lipoprotein cholesterol in human patients. Statins have been shown to decrease Q10 concentrations in skeletal muscle of human patients [59,60,61] and in myocardial tissue in animal models [62]. For the present study, it is fortunate that dogs are rarely prescribed statins and none of the dogs in the study were on statins. Most of the dogs in the CHF group were on standard heart failure treatment, which included combinations of diuretics, phosphodiesterase-3-inhibitor and ACE-i. These pharmaceuticals have not been investigated with regards to their interaction with myocardial Q10 concentrations. We therefore cannot exclude the possibility that any medication used by the dogs could have interfered with myocardial Q10 concentrations.

All the dogs with MMVD in this study were of the breed CKCS while the CON dogs were of other breeds. At our department, owners of CKCS regularly participate in scientific studies in conjunction with their adherence to a national breeding program to prevent MMVD in the breed [63] and this breed is therefore exclusive in the MMVD group. It is currently unknown if depletion of myocardial Q10 concentrations is specifically related to MMVD in the CKCS breed. However, the finding of similar myocardial concentrations of Q10 in CON dogs, which were breeds other than CKCS with MMVD in stages B1 and B2, points towards similar myocardial Q10 concentrations among different breeds in the absence of CHF. We therefore render it unlikely that depleted myocardial Q10 with CHF and MMVD is specific to this breed.

It is important to underline that it is beyond the scope of this study to provide evidence for the use of Q10 supplementation to CKCS diagnosed with MMVD. A recent multicenter, placebo-controlled clinical trial (Q-SYMBIO) including 241 human patients with CHF showed significant clinical improvements in patients receiving 300 mg of Q10 daily over two years compared to placebo [13]. Short-term (3 weeks) treatment with oral Q10 supplementation did not improve clinical parameters of MMVD severity in a cohort of CKCS in a recently published study [64]. A long-term, prospective, placebo-controlled clinical study needs to be done in dogs with MMVD and at risk of developing CHF to determine the clinical relevance of the findings in the present study.

## 5. Conclusions

In conclusion, we present the finding that depleted myocardial concentration of Q10 is associated with CHF in CKCS diagnosed with MMVD. The depletion of Q10 was not associated with a reduction in the mitochondrial density marker citrate synthase activity. No association existed between circulating and myocardial Q10 concentrations in a small number of dogs.

Prospective clinical studies are required to determine if the depletion in myocardial Q10 is a relevant therapeutic target in dogs with MMVD and if it can be restored with supplementation of Q10.

## Figures and Tables

**Table 1:** Background characteristics, standard echocardiographic variables and myocardial concentrations of Q10 (total, reduced, oxidized), oxidation rate, CS activity and Q10/CS in control dogs and dogs with MMVD in different severities.

	CON	B1	B2	C	Overall *p*-Value
N	10	11	5	14	
Breed CKCS/other	0/10	11/0	5/0	14/0	<0.0001 *
Sex Male/Female	4/6	7/4	2/3	8/6	0.72
Body weight (kg)	27.5 (17.1–40.2)	9.0 (8.1–10.1) ^a^	9.1 (9.0–9.9) ^a^	8.7 (7.9–11.4) ^a^	<0.0001 *
BCS (1–9)	5 (5–6)	4 (4–5)	5 (5–5)	5 (4–5)	0.14
MR (no or mild/moderate/severe	8/1/0	0/1/10 ^a^	0/0/5 ^a^	0/0/14 ^a^	<0.0001 *
Murmur (1–6)	0 (0–0)	3 (2–3) ^a^	4 (4–5) ^a,b^	5 (4–5) ^a,b^	<0.0001 *
Age (years)	10.3 (4.0–11.8)	10.8 (8.6–11.3)	12.6 (12.6–12.7)	10.01 (9.3–11.3)	0.15
LA/Ao	1.3 (1.3–1.4)	1.35 (1.3–1.4)	2.0 (1.6–2.1) ^a,b^	2.75 (2.4–3.00) ^a,b,c^	<0.0001 *
LVEDDN	1.4 (1.30–1.60)	1.6 (1.5–1.7)	1.9 (1.9–2.1) ^a,b^	2.3 (1.9–2.6) ^a,b^	<0.0001 *
FS (%)	33.6 (33.0–38.8)	35.5 (29.4–49.9)	40.3 (39.8–43.2)	45.4 (42.3–47.5)	0.091
Total Q10, myocardium, (µg/mg)	2.8 (1.64–4.88)	2.76 (2.10–4.81)	3.85 (3.13–4.46)	1.54 (1.36–1.94) ^a,b,c^	0.0035 *
Ubiquinone, myocardium, (µg/mg)	0.97 (0.56–1.47)	1.9 (0.82–2.08)	1.2 (0.96–1.46)	0.59 (0.44–0.66) ^a,b,c^	0.0008 *
Ubiquinol, myocardium, (µg/mg)	1.84 (1.07–3.68)	1.7 (1.06–2.73)	2.89 (1.93–3.00)	0.96 (0.76–1.33) ^a,b,c^	0.029 *
Oxidation rate (%)	35.5 (32.4–42.3)	42.2 (34.1–49.4)	38.2 (32.7–38.3)	36.6 (32.0–39.9)	0.076
CS μmol/g/min (*n* = 34)	120.7 (109.6–138.5) *n* = 10	127.1 (121.8–130.7) *n* = 7	135.1 (121.9–135.5) *n* = 5	119.3 (111.95–128.3) *n* = 12	0.79
Q10/CS	0.017 (0.014–0.042)	0.034 (0.014–0.044)	0.033 (0.029–0.049)	0.014 (0.0099–0.016) ^a,b,c^	0.0077 *

Myocardial Q10 concentrations were successfully measured in included 40 dogs. CS activity was analyzed in 34 dogs while in the remaining dogs there was not sufficient tissue sample left for this analysis. Echocardiographic assessment of MR was missing for one dog (CON). Abbreviations: ACVIM: American College of Veterinary Internal Medicine; CS: citrate synthase; FS: Fractional Shortening; LA/Ao: Left atrial to aortic root ratio; LVEDDN: Left ventricular internal diameter in diastole normalized for body weight; MR: mitral regurgitation. Data are presented as median (interquartile range). N = 40 unless stated otherwise. * denotes statistically significant difference between groups. *p*-values were calculated using one-way ANOVA with post hoc *t*-test. Categorical parameters were tested using Fisher’s exact test. ^a^: Different from CON, ^b^: Different from B1, ^c^: different from B2.

## Data Availability

The data presented in this study are available on request from the corresponding author.
